# Inflammasome Dynamics in the High-Risk Clone ST235 with Altered Pyoverdine Structures

**DOI:** 10.3390/ijms27188040

**Published:** 2026-09-09

**Authors:** Zeynep Gülçe Tanyolaç

**Affiliations:** Department of Microbiology and Immunology, University of Massachusetts Chan Medical School, Worcester, MA 01655, USA; zeynepgulce94@gmail.com

**Keywords:** inflammasome, macrophages, cell death, *Pseudomonas aeruginosa*, ST235 high risk clone, pyoverdine, macrophage polarization

## Abstract

*Pseudomonas aeruginosa* ST235 isolates drive a distinct macrophage inflammatory program characterized by IL-1β axis dominance and suppression of canonical NF-κB-associated cytokine responses. In THP-1-derived macrophages, ST235 isolates induce robust inflammasome activation, resulting in elevated IL-1β production and altered cell death responses, while eliciting markedly lower TNFα, IL-6, IL-8, and IL-10 responses than non-ST235 isolates. In contrast, nonST235 isolates preferentially stimulate NF-κB-dependent cytokine production with weaker inflammasome activation. Mechanistically, ST235 infection suppresses NF-κB signaling and reduces caspase-1 expression, whereas caspase-8 remains unchanged, indicating that increased IL-1β production can be due to alternative inflammasome pathways or posttranscriptional modifications. Rapid macrophage death further restricts overall cytokine production, reinforcing an IL-1β-dominant inflammatory profile. ST235 isolates also selectively reprogram M2-polarized macrophages toward an M1 phenotype, as demonstrated by increased CD86 expression, while both isolate groups promote M1 polarization in unpolarized macrophages. Importantly, pyoverdine purified from ST235 isolates reproduced aspects of these responses by inducing IL-1β production and cytotoxicity more effectively than pyoverdine from non-ST235 isolates, supporting a potential contribution of ST235-derived pyoverdine to the IL-1β-dominant inflammatory phenotype. Collectively, these findings suggest that ST235 isolates modulate macrophage immune responses through suppression of NF-κB-associated responses and promotion of IL-1β-dominant inflammation, providing insights into the virulence of this high-risk clone and highlighting potential therapeutic targets. Significant statement: ST235 isolates of *P. aeruginosa* uniquely modulate macrophage immune responses by robustly activating inflammasome signaling, inducing IL-1β expression while suppressing other cytokines, and driving macrophage polarization toward a pro-inflammatory M1 phenotype. These findings highlight distinct pathogenic strategies of ST235 isolates that may inform targeted therapeutic interventions.

## 1. Introduction

*Pseudomonas aeruginosa* is a Gram-negative opportunistic pathogen and a major cause of hospital-acquired infections, particularly among critically ill and immunocompromised patients. Its intrinsic resistance to multiple antimicrobial classes and remarkable capacity to acquire additional resistance determinants have contributed to the emergence and global dissemination of multidrug-resistant (MDR) high-risk clones. Among these, ST235 is a globally prevalent and clinically important clone that has been associated with multidrug resistance and severe infections, posing a significant therapeutic challenge [1]. The pathogenicity and persistence of *P. aeruginosa* are mediated by a diverse repertoire of virulence factors, including siderophores, which play a critical role in iron acquisition under the iron-limited conditions encountered within the host [2]. *P. aeruginosa* produces two major siderophores, pyochelin and pyoverdine. Pyoverdine has a higher affinity for iron than pyochelin and functions as a key iron-chelating molecule, scavenging iron from the environment and facilitating its transport into bacterial cells. *P. aeruginosa* can produce structurally distinct pyoverdine types, including type I and type II pyoverdines, and their production can vary depending on iron availability and interactions with other microorganisms [3]. Beyond its essential role in iron acquisition, pyoverdine has also been implicated in bacterial virulence and modulation of host immune responses, suggesting that this siderophore may contribute to the complex interactions between *P. aeruginosa* and the host during infection [4]. Therefore, understanding the relationship between clinically relevant *P. aeruginosa* lineages, siderophore-mediated iron acquisition, and host immune responses may provide important insights into the mechanisms underlying the pathogenicity and persistence of high-risk clones such as ST235.

The innate immune system is the first line of host defense, which results in the lysis of infected phagocytes through pyroptosis and the secretion of the IL-1ß cytokine family following the assembly of intracellular inflammatory platforms called inflammasomes. Inflammasomes play a significant role in *P. aeruginosa* infections. The Nod-like receptor protein 3 (NLRP3) and Nod-like receptor containing a CARD domain 4 (NLRC4) pathways are the most critical inflammasome responses in *P. aeruginosa*-related respiratory infections [5].

*P. aeruginosa* undergoes genetic adaptations to evade the innate immune system. To achieve this, the bacteria can gain traits such as the secretion of more virulence factors, adaptation to biotic or abiotic changes in the environment, and enhanced nutrient acquisition. Changes in pyoverdine molecules make the bacteria more virulent [6]. However, no study has systematically compared the immune responses elicited by ST235 and non-ST235 isolates of *P. aeruginosa*. In this study, we aimed to characterize the differential effects of ST235 and non-ST235 isolates on macrophage inflammatory responses, with particular emphasis on NF-κB signaling, inflammasome-associated pathways, cytokine production, and macrophage polarization. Therefore, this study aimed to investigate whether differences in pyoverdine derived from ST235 and non-ST235 isolates contribute to the distinct inflammatory phenotypes observed. We hypothesized that ST235 isolates and their pyoverdine would differentially modulate macrophage responses, resulting in an IL-1β-dominant inflammatory phenotype and altered cell death responses compared with non-ST235 isolates.

## 2. Results

### 2.1. ST235 and nonST235 P. aeruginosa Show Similar Inhibition Rates, but ST235-Infected Macrophages Exhibit Higher Viability

**Figure 1:**
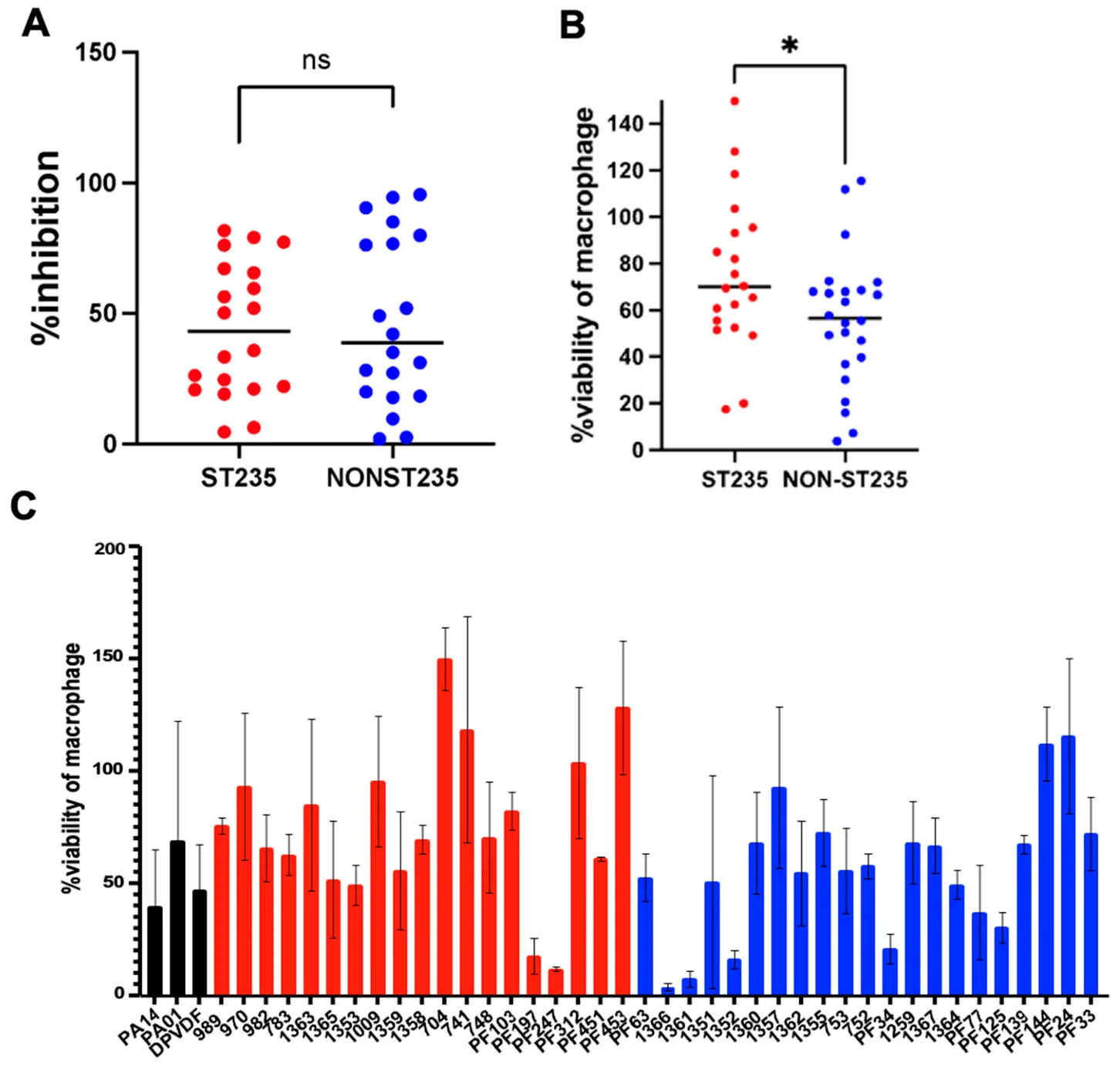
ST235 isolates are associated with higher macrophage viability than nonST235 isolates. THP-1-derived macrophages were seeded at 500,000 cells/well and infected with the indicated ST235 and non-ST235 isolates of *P. aeruginosa*, PA14, PAO1, and ΔPVDF (10 MOI). Comparison of inhibition rate for ST235 and non-ST235 isolates of *P. aeruginosa* (**A**). Cell viability of all isolates of *P. aeruginosa* (**B**). Viability percentage of THP-1-derived macrophages after being infected with ST235 and non-ST235 isolates of *P. aeruginosa* (**C**). Student’s *t*-tests were used to compare ST235 and non-ST235 isolates for cell viability. Mean ± SD of triplicates of a representative isolate, or a pool of 3 experiments, is shown. (* *p* < 0.05).

THP-1-derived macrophages were used and infected with PA14, PAO1, dPVDF, and clinical isolates. In terms of bacterial inhibition rate, there was no difference between ST235 (mean of ST235:46.68) and non-ST235 (mean of non-ST235: 43.95) isolates (Figure 1A) (*p* = 0.7683).

PA14 and PAO1 are commonly used reference strains for pathogenesis studies, which were originally isolated from burn/wound patients. Thus, after THP-1-derived macrophages were infected with PA14 and PAO1 isolates, the percentage of bacterial inhibition for PA14 (72%) and PAO1 (68%) was nearly the mean of ST235 (mean of ST235:75.3) and non-ST235 (mean of non-ST235: 55.68) isolates of *P. aeruginosa* (Figure 1B).

However, the cell death assay showed that THP-1-derived macrophages were significantly more viable after infection with ST235 (mean of ST235:75.3) isolates than non-ST235 (mean of non-ST235: 55.68) isolates (Figure 1B,C) (*p* = 0.0444).

### 2.2. ST235 Isolates of P. aeruginosa Induce Inflammasome-Related Cytokine IL-1β More Than nonST235 Isolates

**Figure 2:**
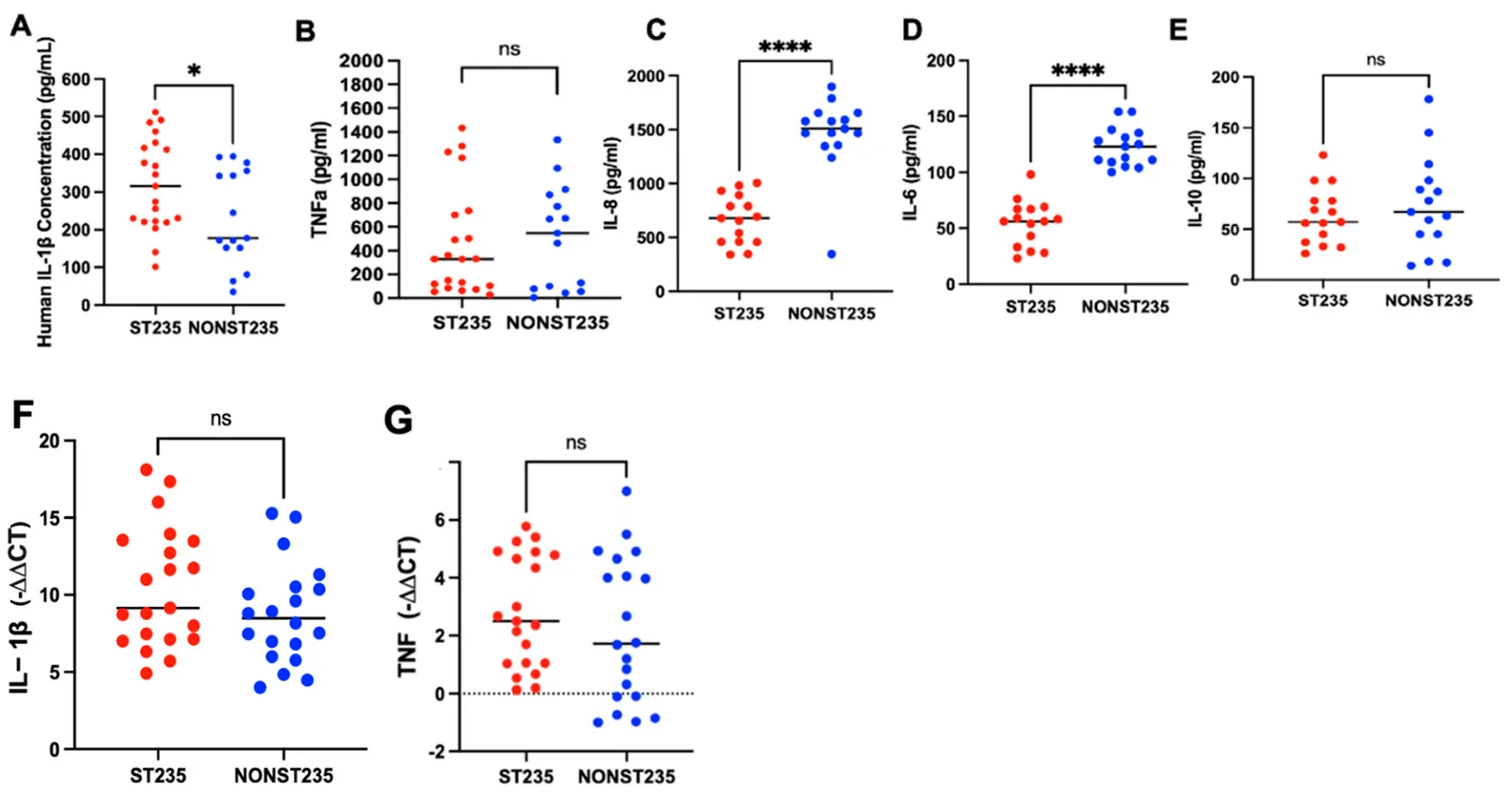
ST235 isolates of *P. aeruginosa* induced IL-1β production but not at the mRNA level. THP-1-derived macrophages were seeded at 500,000 cells/well and infected with the indicated ST235 and non-ST235 isolates of *P. aeruginosa* or PA14, PAO1, and ΔPVDF (10 MOI). Cytokine production was measured via ELISA at 3 h postinfection. (**A**) Comparison of ST235 and non-ST235 isolates of *P. aeruginosa* for IL-1β (**B**), TNFα (**C**), IL-8 (**D**), IL-6 (**E**), and IL-10. RNA was extracted, and cDNA was synthesized from THP-1-derived macrophages infected with ST235 and non-ST235 isolates. qPCR was conducted on samples targeting genes for various cytokines, and mRNA levels were normalized to β-actin (**F**,**G**). Student’s *t*-tests were used to compare the two groups. Mean ± SD of triplicates of a representative, or a pool of 3 experiments, is shown. (* *p* < 0.05, **** *p* < 0.0001).

We assessed cytokine production by macrophages following infection with ST235 or non-ST235 isolates to investigate the differences in cellular responses following infection. IL-1β production was higher in macrophages infected with ST235 isolates compared with those infected with non-ST235 isolates (Figure 2A). Although TNF-α and IL-10 levels did not differ significantly between the groups, IL-6 and IL-8 levels were higher in macrophages infected with non-ST235 isolates compared with those infected with ST235 isolates. These findings indicate differential cytokine responses to ST235 and non-ST235 isolates, with ST235 infection associated with increased IL-1β production, whereas non-ST235 infection was associated with higher IL-6 and IL-8 production (Figure 2B–E).

Next, we aimed to determine whether the observed difference in cytokine production was due to variations at the mRNA level or disruptions in the cell signaling pathways. We then checked mRNA levels of IL-1β and TNFα through quantitative reverse transcription polymerase chain reaction (qRT-PCR) analysis. We did not observe any difference between ST235 and nonST235 after 3 h of infection (Figure 2F,G), suggesting that any differences in measured cytokines in the supernatant of infected cells may be due to interruptions in the cell signaling process or post-transcriptional regulation.

### 2.3. ST235 Isolates Reduced Caspase-1 Activity of Macrophages Compared to nonST235 Isolates

**Figure 3:**
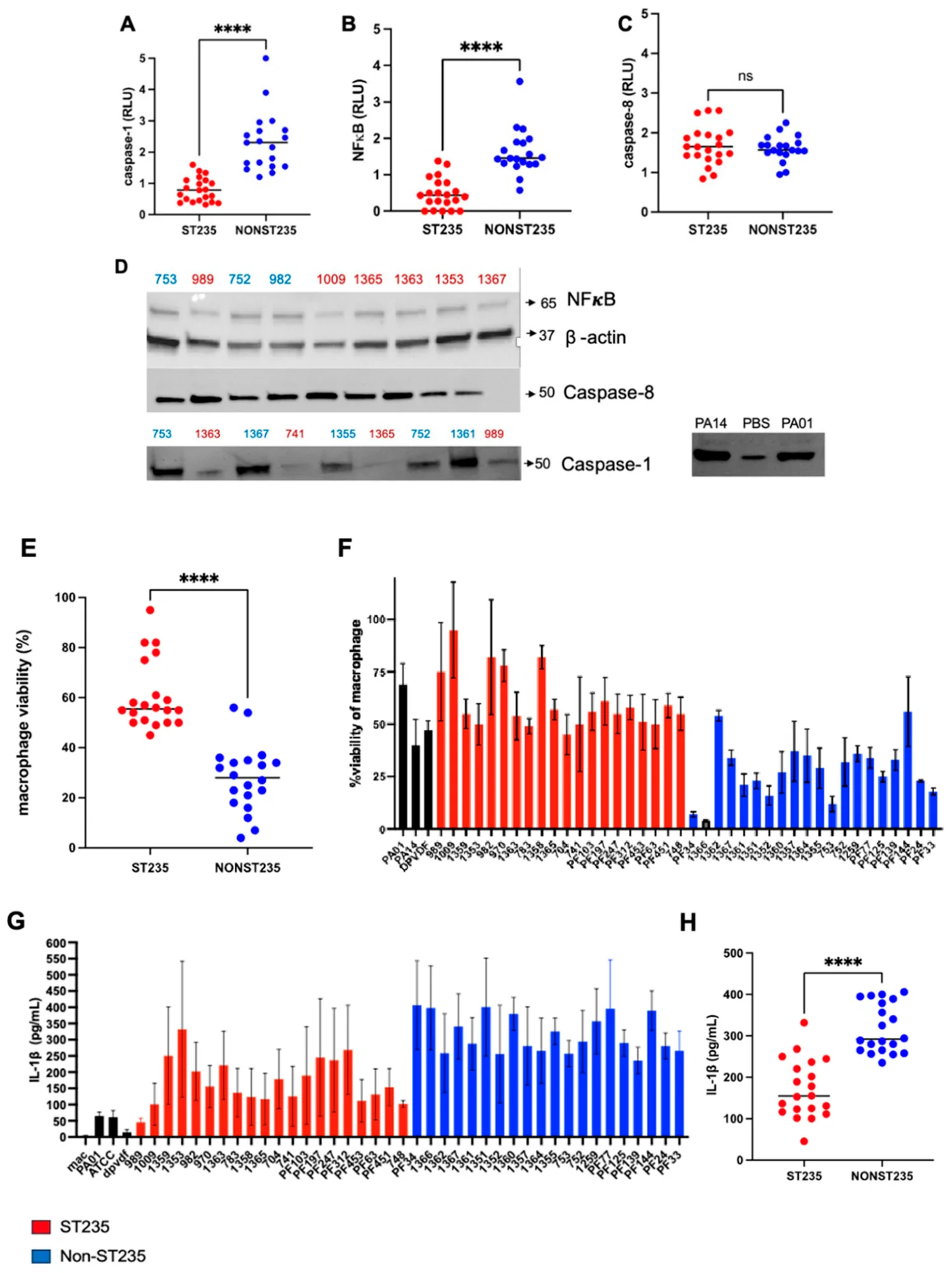
Role of caspase-1 in cell death and IL-1β secretion after being infected with ST235 and non-ST235 isolates of *P. aeruginosa.* Densitometric analysis of Western blots shown in D was conducted (**A**–**C**). THP-1-derived macrophages were seeded at 500,000 cells/well in a 24-well plate. At 3 h post-infection (10 MOI) with the indicated ST235 and non-ST235 isolates of *P. aeruginosa*, Western blotting was performed on cell extracts using specific antibodies shown in (**D**). Cell viability of all isolates of *P. aeruginosa* after being infected with defcaspase-1 THP-1 cells (**E**). Comparison of cell viability for ST235 and non-ST235 isolates of *P. aeruginosa* (**F**). IL-1β production was measured via ELISA at 3 h postinfection (**G**). Comparison of IL-1β production for ST235 and non-ST235 isolates of *P. aeruginosa* (**H**) (**** *p* < 0.0001).

We therefore checked caspase-1 involvement after infection with ST235 and non-ST235 isolates. Pro-Caspase-1 protein levels were reduced in macrophages after being infected with ST235 isolates (Figure 3A) (mean of ST235 and non-ST235, respectively; 0.7983 and 2.329 RLU; *p* value: <0.0001). Similarly, activity of NFκB proteins after infection with ST235 (mean of ST235 0.4816; *p* value: <0.0001) isolates was significantly less than non-ST235 (mean of non-ST235: 1.624 RLU; *p* value: <0.0001) isolates (Figure 3B). We also checked the abundance of pro-caspase-8 protein since caspase-8 can contribute to the production of IL-1β through both direct and indirect mechanisms, highlighting its role in inflammatory signaling pathways. However, we did not observe any difference between ST235 and non-ST235 (mean of ST235 and non-ST235, respectively; 1.672 and 1.605; *p* value: 0.6037) isolates in pro-caspase-8 protein levels (Figure 3C). Interestingly, ST235 infection was associated with reduced pro-caspase-1 protein levels despite increased IL-1β secretion, suggesting that the elevated IL-1β response may involve mechanisms beyond canonical caspase-1-dependent regulation, which is further discussed below.

We next examined the role of caspase-1 in cell death and IL-1β secretion during infection with ST235 and nonST235 isolates of *P. aeruginosa* using THP-1-defCASP1 cells. Similar to wild-type THP-1 cells, the nonST235 (mean of nonST235: 28% viability) *P. aeruginosa* isolates induced more cell death in THP-1 cells in comparison to ST235 isolates mean of ST235: 60% viability) (*p* value: <0.0001) (Figure 3E,F). The results showed that cell death appeared to be primarily mediated by an independent of caspase-1 mechanism, regardless of whether the cells were infected with ST235 or non-ST235 isolates.

The IL-1β production was lower in THP-1-defCASP1 cells after infection with ST235 (mean of ST235: 171 pg/mL) isolates compared to non-ST235 (mean of non-ST235: 318 pg/mL) isolates, in contrast to wild-type THP-1 cells (*p* value: 0.001) (Figure 3G,H). Therefore, while caspase-1 likely does not play a major role in cell death during infection with ST235 isolates of *P. aeruginosa*, canonical inflammasome activation appears to play a role in IL-1β maturation and secretion.

### 2.4. M2 Macrophages Polarized to M1 Macrophages After Being Infected with ST235 Isolates of P. aeruginosa

**Figure 4:**
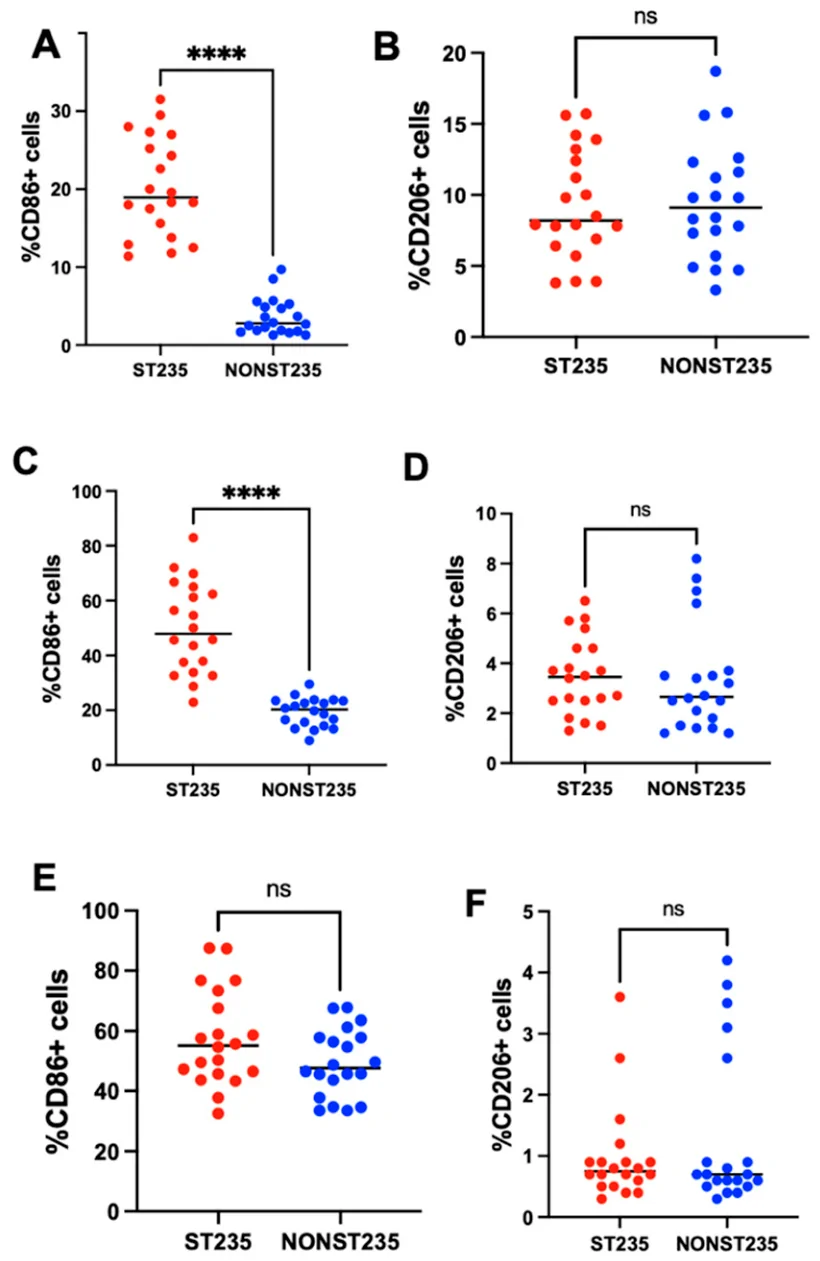
Effect of ST235 and non-ST235 isolates of *P. aeruginosa* on macrophage polarization. THP-1-derived macrophages were seeded at 500,000 cells/well and polarized to M1 and M2 macrophages with INFγ and IL-10, respectively. Cells were stained with CD86 and CD206. Comparison of CD86+ cells in ST235 and non-ST235 isolates after infection with M2-polarized macrophages (**A**). CD206+ cells in ST235 and non-ST235 isolates after infection with M2-polarized macrophages (**B**). CD86+ cells in ST235 and non-ST235 isolates after infection with M1-polarized macrophages (**C**). CD206+ cells in ST235 and non-ST235 isolates after infection with M1-polarized macrophages (**D**). CD86+ cells in ST235 and non-ST235 isolates after infection with M0 macrophages (**E**). CD206+ cells in ST235 and non-ST235 isolates after infection with M0-polarized macrophages (**F**). Student’s *t*-tests were used to compare the two groups; a pool of 3 experiments is shown. (**** *p* < 0.0001).

THP-1-derived macrophages were polarized into M1 and M2 macrophages, then ST235 and non-ST235 isolates were added to the macrophages. After 3 h, macrophages were stained for CD206 and CD86 surface markers. We observed an increase in the percentage of CD86+ M2-polarized macrophages after 3 h of infection with ST235 isolates (mean of CD86+ M2 macrophages for ST235 and nonST235, respectively: 20.3 and 3.68). On the other hand, we did not observe as much of an increase in the percentage of CD86+ M1-polarized macrophages infected with nonST235 isolates (mean of CD86+ M1 macrophages for ST235 and nonST235, respectively: 50.11 and 19.34) (*p* value for M1 and M2 polarized macrophages, respectively: 0.001 and 0.001) (Figure 4A,B).

We did not observe any difference in terms of CD206+ cells between ST235 and nonST235 isolates after being infected with M1-polarized (mean of CD86+ M1 macrophages for ST235 and nonST235, respectively: 3.490 and 3.355) and M2-polarized macrophages (mean of CD86+ M2 macrophages for ST235 and nonST235, respectively: 9.325 and 9.495) (*p* value for M1 and M2 macrophages, respectively: 0.8213 and 0.8932). Also, there was no difference in CD206+CD86+ cells after infection with M1- or M2-polarized cells (Figure 4D,E).

Then, THP-1-derived macrophages were exposed to ST235 and nonST235 isolates of *P. aeruginosa* for 3 h. Macrophages were stained with CD86 and CD206 surface markers without being polarized into M1 or M2 macrophages. The results showed that macrophages skewed into M1 macrophages when they are infected with both of the isolates (mean of CD86+ M0 macrophages for ST235 and nonST235, respectively: 57.57 and 49.32) and (mean of CD206+ M0 macrophages for ST235 and nonST235, respectively: 0.9850 and 1.320) (*p* value for CD86+ cells and CD206+ cells, respectively: 0.0660 and 0.3323) (Figure 4E,F).

### 2.5. Pyoverdine Secreted by ST235 İnduces More Cell Death and IL-1β Cytokine but Reduced TNFα Compared to Non-ST235 Isolates of P. aeruginosa

To further characterize the structural features of pyoverdine, the molecule was extracted from PAO1, one ST235 isolate, and one non-ST235 isolate and analyzed by NMR spectroscopy. Following lyophilization, samples were subjected to comparative 2D ^1H–^13C HMQC analysis. No detectable differences were observed between pyoverdine derived from PAO1 and the nonST235 isolate. In contrast, comparison of ST235-derived pyoverdine with PAO1 revealed distinct spectral alterations. Detailed examination of the HMQC spectra identified two major differences. First, the cross-peak at 3.40 ppm (^1H)/45.80 ppm (^13C), present in PAO1-derived pyoverdine, was absent in the ST235 sample. This signal corresponds to the C–H bond in the OHOrn7 δ-proton region, which exhibited a pronounced chemical shift unique to the ST235 isolate. This shift may result either from an increased population of the chromophore region interacting with iron in solution or from structural changes in the ornithine moiety that create steric effects on the surrounding atoms. Second, the cross-peak at 8.06 ppm (^1H)/135.42 ppm (^13C) in PAO1 shifted to 8.12 ppm (^1H)/135.24 ppm (^13C) in the ST235 isolate, corresponding to the chromophore region (Chr C9). Overlay of the 2D ^1H–^13C HMQC spectra from PAO1 (red) and ST235 (green) highlights these differences (Figure S1, Supplementary Materials).

**Figure 5:**
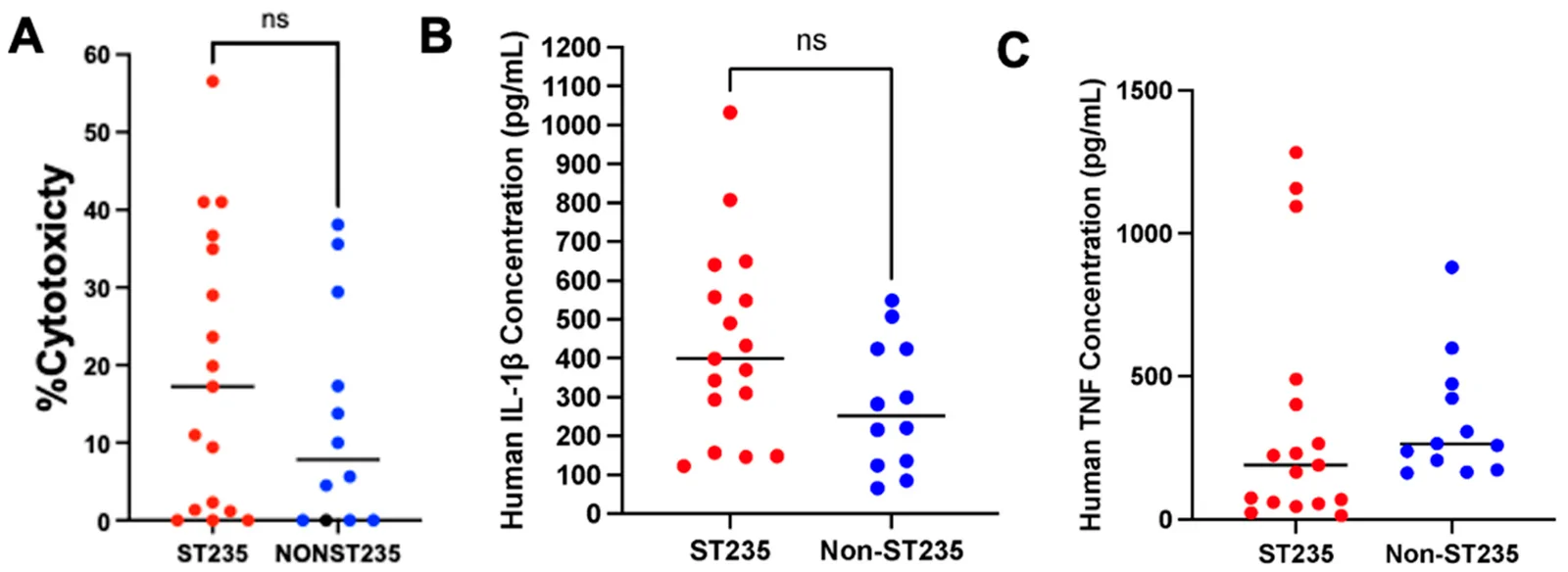
Effect of extracted pyoverdine from ST235 or non-ST235 isolates of *P. aeruginosa* on THP-1-derived macrophages. THP-1 derived macrophages were seeded at 500,000 cells/well and infected with the extracted pyoverdine from ST235 and nonST235 isolates of *P. aeruginosa*. Cell cytotoxicity was measured via AlamarBlue assay. Comparison of the cytotoxicity of extracted pyoverdine from ST235 and non-ST235 isolates of *P. aeruginosa* (**A**). Cytokine production was measured via ELISA at 3 h post-infection. IL-1β (**B**,**C**) and TNFα production. Mann–Whitney U tests were used to compare the two groups; a pool of 3 experiments is shown.

To assess the potential functional relevance of the observed structural variations, THP-1-derived macrophages were exposed to pyoverdine extracted from ST235 and non-ST235 isolates. Pyoverdine derived from ST235 isolates showed a higher median cytotoxicity (17%) than pyoverdine derived from non-ST235 isolates (8%); however, this difference was not statistically significant (*n* = 30, *p* = 0.3601) (Figure 5A). We next examined cytokine production by THP-1-derived macrophages following treatment with pyoverdine extracted from ST235 and non-ST235 isolates of *P. aeruginosa*. ST235-derived pyoverdine showed a trend toward higher IL-1β production (median: 400 pg/mL) compared with nonST235-derived pyoverdine (median: 252 pg/mL), although this difference did not reach statistical significance (*p* = 0.0606, *n* = 30) (Figure 5B). IL-1β production also showed a trend toward higher levels following treatment with ST235-derived pyoverdine compared with the laboratory reference strains PA14 and PAO1. In contrast, TNFα production showed a trend toward lower levels following treatment with ST235-derived pyoverdine (median: 191 pg/mL) compared with non-ST235-derived pyoverdine (median: 263 pg/mL); however, this difference was not statistically significant (*p* = 0.1986) (Figure 5C). Overall, although these findings suggest potential differences in the biological effects of pyoverdine derived from ST235 and non-ST235 isolates, the lack of statistical significance indicates that the observed trends should not be interpreted as evidence that pyoverdine alone is responsible for the phenotypic differences between ST235 and non-ST235 isolates.

## 3. Discussion

*P. aeruginosa* infects the lungs of immunocompromised individuals and adapts to the airway environment [7,8,9,10]. Among all isolates of *P. aeruginosa*, the ST235 isolate is indeed one of the most prevalent of the so-called ‘high-risk’ clones. This clone is associated with poor clinical outcomes, in part due to its multi-level and high-level antibiotic resistance. This makes it particularly challenging to treat infections caused by this strain [11,12,13,14,15].

In the lungs of CF patients, *P. aeruginosa* isolates evolve and exhibit a reduced virulence phenotype to evade host immunity. Studies on CF patients have shown that inflammatory cytokines, such as TNFα and IL-8, are often elevated in the bronchoalveolar lavage fluid of those with ongoing infections [16]. However, there are no studies specifically focused on the macrophage response to ST235 isolates. These findings reveal for the first time an inverse relationship between macrophage cell death and inflammasome activation in response to ST235 isolates. In contrast, pro-inflammatory cytokines such as TNFα, IL-6, and IL-8, as well as the anti-inflammatory cytokine IL-10, were elevated in macrophages infected with non-ST235 isolates. Consistent with our findings, Phuong et al. reported an inverse relationship between cell death and IL-1β production in chronic versus early *P. aeruginosa* isolates from cystic fibrosis patients [17].

Our findings show that ST235 and nonST235 isolates of *P. aeruginosa* elicit distinct immune responses in macrophages, as reflected by differences in cytokine production and inflammasome-associated responses. The observed differences may be related, at least in part, to the altered type 2 pyoverdine produced by ST235 isolates. Consistent with this interpretation, experiments with purified pyoverdine did not reveal statistically significant differences between ST235 and non-ST235 isolates. Therefore, the other strain-specific bacterial factors may also contribute to the observed macrophage responses. Several studies suggest that modulation of inflammasome activation and cell death by pyoverdine may contribute to immune evasion and persistence of *P. aeruginosa* isolates [18,19,20,21,22]. In line with this concept, the increased IL-1β production together with altered NFκB signaling observed in ST235-infected macrophages is consistent with enhanced inflammasome engagement and a shift in inflammatory programming. While the precise molecular mechanisms require further delineation, our findings support the notion that pyoverdine-associated alterations may influence inflammasome-related pathways and macrophage survival. In contrast, nonST235 isolates induced higher levels of TNFα, IL-6, and IL-8, consistent with enhanced NFκB activity observed in these infections. This pattern suggests that nonST235 isolates promote a broader NFκB–driven inflammatory response, whereas ST235 isolates preferentially skew macrophage signaling toward an IL-1β-dominant axis. Additionally, there might be regulatory mechanisms within macrophages that modulate the balance between cell death and inflammasome activation. A study comparing different clinical isolates of *P. aeruginosa* showed that these regulatory mechanisms involve specific signaling pathways or regulatory proteins, which prioritize inflammasome responses over cell death under certain conditions [17]. From the bacteria’s perspective, ST235 isolates may have evolved strategies to manipulate host cell responses to enhance their survival and proliferation. In light of the study by Karki et al. showed that suppressing cell death allows clinical isolates of *P. aeruginosa* from CF patients to avoid premature clearance by the immune system, allowing them to persist and replicate within the host [23]. Conversely, the host immune system might be optimizing its response to maximize pathogen clearance while minimizing tissue damage. By favoring inflammasome activation over cell death, macrophages can effectively release pro-inflammatory cytokines to recruit other immune cells without undergoing excessive pyroptosis, which can be detrimental to the host.

THP-1-derived macrophages were exposed to pyoverdine extracted from ST235 and nonST235 *P. aeruginosa* isolates. Consistent with the trends observed in the bacterial infection experiments, macrophages treated with pyoverdine extracted from ST235 isolates showed a trend toward higher IL-1β production compared with those treated with pyoverdine from non-ST235 isolates; however, this difference did not reach statistical significance. Previous studies have shown that pyoverdine, the siderophore produced by *P. aeruginosa*, can influence host immune responses. However, studies specifically investigating the immunomodulatory effects of extracted pyoverdine from *P. aeruginosa* clinical isolates remain limited. Our findings suggest a potential association between differences in pyoverdine and the distinct macrophage responses observed, although the underlying mechanism remains to be determined. The altered pyoverdine structure identified in ST235 isolates may contribute to these differences, but this possibility requires further investigation. A study showed that pyoverdine-deficient *P. aeruginosa* strains exhibited reduced virulence, supporting a role for pyoverdine in host–pathogen interactions [24,25]. Importantly, endotoxin removal or endotoxin testing was not performed in the present study. Therefore, the possibility of residual LPS contamination during pyoverdine extraction cannot be excluded and may have contributed to the observed immune responses. This limitation should be considered when interpreting the effects of extracted pyoverdine, and further studies using endotoxin-controlled preparations are needed to determine the specific contribution of pyoverdine to the observed macrophage responses. Caspase-1 is a key enzyme involved in inflammasome-mediated maturation and release of pro-inflammatory cytokines such as IL-1β and IL-18 [26,27,28,29,30,31]. In this study, macrophages infected with ST235 isolates exhibited lower pro-caspase-1 protein levels compared with those infected with non-ST235 isolates. The ST235 strain of *P. aeruginosa* might involve alternative inflammasome-associated mechanisms that contribute to IL-1β production despite lower pro-caspase-1 protein levels. In particular, *P. aeruginosa* has been shown to induce IL-1β maturation independently of caspase-1, including through bacterial proteases such as LasB, and this mechanism has been demonstrated in macrophage models. In addition, caspase-8 and neutrophil-derived proteases have been implicated in alternative IL-1β maturation pathways, indicating that IL-1β release does not necessarily correlate directly with cellular caspase-1 protein abundance or cell death [32,33]. Notably, caspase-1-independent IL-1β processing has also been reported during *P. aeruginosa* infection through neutrophil serine proteases. Although our data do not establish the involvement of these alternative pathways in ST235-infected macrophages, these mechanisms provide possible explanations for the increased IL-1β secretion observed despite reduced caspase-1 protein levels. Further studies assessing IL-1β processing and alternative inflammatory proteases will be required to determine the underlying mechanism. This possibility may contribute to the higher IL-1β production observed in ST235-infected cells. Alternatively, non-ST235 isolates may rely more strongly on the classical caspase-1-dependent pathway for IL-1β production. Consistent with this possibility, IL-1β production in non-ST235-infected cells was significantly reduced in the absence of caspase-1. The higher IL-1β production observed in ST235-infected cells despite lower pro-caspase-1 protein levels suggests that IL-1β production in response to ST235 may involve caspase-1-independent mechanisms. This alternative mechanism may be less prominent in non-ST235 isolates, potentially contributing to the lower IL-1β production observed in the absence of caspase-1.

Another possible explanation for the difference in pro-caspase-1 protein abundance is the altered pyoverdine produced by ST235 isolates. The lower pro-caspase-1 protein levels observed in macrophages infected with ST235 isolates may reflect differential modulation of host immune responses. Consistent with this possibility, a study comparing T3SS-mutated and wild-type *P. aeruginosa* isolates in a corneal infection model reported suppression of caspase-1-associated responses following infection with the T3SS-mutated isolate [34]. However, the mechanisms underlying the lower pro-caspase-1 protein abundance observed in ST235-infected macrophages remain unclear.

Pyoverdine represents one potential factor that may contribute to this differential host response. Pyoverdine can chelate iron from the host environment, and iron availability is important for various cellular processes, including immune responses [6,35,36,37]. By altering iron availability, pyoverdine may influence cellular signaling pathways associated with inflammasome responses. In addition, previous studies have shown that pyoverdine can enter macrophages and affect mitochondrial function, potentially increasing oxidative stress through disruption of intracellular iron homeostasis [25,38,39]. These effects provide a possible mechanism through which differences in pyoverdine production or activity could influence macrophage responses to ST235 and nonST235 isolates. However, our experiments with purified pyoverdine did not reveal statistically significant differences between ST235 and nonST235 isolates. Therefore, the observed differences in pro-caspase-1 protein abundance cannot be attributed solely to pyoverdine, and other strain-specific bacterial factors may also contribute to the differential host response. Further studies will be required to determine whether and how the altered pyoverdine structure of ST235 isolates contributes to the regulation of inflammasome-associated responses. Moreover, there are studies showing that isolates of *P. aeruginosa* can reduce pro-caspase-1 protein levels [18,40,41]. Specifically, *P. aeruginosa* secretes various effector molecules, such as ExoU, through its type III secretion system, which can modulate host immune responses [42]. ExoU, a potent phospholipase, has been shown to inhibit caspase-1 activation, thereby preventing inflammasome activation and subsequent pyroptosis in host cells. This inhibition helps the bacteria evade the host immune system and promotes bacterial survival and persistence within the host [34]. In this study, we found no differences in pro-caspase-8 protein levels between macrophages infected with ST235 and non-ST235 isolates from *P. aeruginosa*. The lack of difference in pro-caspase-8 protein levels between ST235 and non-ST235 isolates suggests that both types of isolates might not modulate the extrinsic apoptotic pathway significantly. Caspase-8 is primarily involved in apoptosis rather than pyroptosis or inflammasome activation, which are more closely associated with caspase-1. Therefore, the modulation of caspase-1, which is more directly linked to inflammasome activation and pyroptosis, could be a more critical target for these bacterial isolates. Additionally, this finding implies that the differences in immune response and cell death mechanisms are more likely driven by pathways involving caspase-1 rather than those involving caspase-8. However, contrary to our results showing no difference in pro-caspase-8 protein levels, previous studies have demonstrated that pyoverdine reduces caspase-8 activation, leading to decreased IL-1β secretion during Listeria monocytogenes and Acinetobacter baumannii infections [43,44,45]. This contradiction could be due to differences in bacterial types because even different isolates of *P. aeruginosa* activate distinct pathways.

This strain-dependent difference may also be considered in the context of the two-step regulation of inflammasome signaling. Inflammasome responses are initiated by a priming signal, commonly involving NFκB-dependent transcription of pro-inflammatory and inflammasome-related genes, followed by an activation signal that promotes inflammasome assembly and caspase-1 activation [46]. Previous studies have demonstrated that distinct *P. aeruginosa* clinical isolates can differentially regulate these inflammatory responses. In particular, early clinical isolates have been shown to induce stronger inflammasome signaling, cell death, and IL-1β production, whereas chronic isolates preferentially induced NFκB-associated cytokines, including TNF, IL-6, and IL-8, suggesting that strain-specific bacterial phenotypes can differentially influence inflammasome-associated responses [17]. Consistent with this concept, the *P. aeruginosa* virulence factor ExoY has been shown to modulate both NF-κB and caspase-1 responses, demonstrating that bacterial virulence factors can influence distinct components of inflammasome-associated signaling [47]. Furthermore, recent work demonstrated that the inflammasome pathway engaged during *P. aeruginosa* infection can vary according to both the bacterial virulence factors and the host-cell context; whereas PAO1-induced IL-1β secretion in macrophages was dependent on NLRC4, PAO1 induced NLRP3-dependent IL-1β secretion in neutrophils through the activity of the T3SS effector ExoS [48]. In this study, the stronger NFκB-associated cytokine response observed in macrophages infected with non-ST235 isolates may reflect a more pronounced priming response, whereas the more prominent IL-1β response observed following infection with ST235 isolates producing altered pyoverdine may indicate a greater contribution of the downstream inflammasome activation phase. Given the altered pyoverdine characteristics of the ST235 isolates examined in this study, these strain-dependent differences may partly reflect differential effects of virulence-associated factors on host inflammatory signaling. Thus, the contrasting phenotypes induced by ST235 and non-ST235 isolates may represent differences in the relative balance between inflammasome priming and activation rather than simply differences in the overall magnitude of inflammasome signaling.

Macrophage polarization is important to fight the pathogen [49,50,51]. The results of this study for the first time demonstrate a significant phenotypic shift in macrophage polarization following infection with ST235 isolates, while macrophages infected with non-ST235 isolates did not show as much change as ST235 isolates. Specifically, macrophages that were initially polarized to an M2 phenotype exhibited markers characteristic of M1 macrophages upon exposure to these pathogenic isolates. This observation aligns with previous studies indicating that bacterial infections can influence macrophage polarization, thereby impacting the host’s immune response and the progression of infection [49]. This study provides compelling evidence that ST235 isolates can induce a phenotypic shift from M2 to M1 macrophages, with significant implications for the immune response and pathogenesis of infection.

Several limitations of the present study should be acknowledged. First, our experiments were performed primarily using THP-1-derived macrophages, which are an immortalized cell model and may not fully recapitulate the phenotype and responses of primary human macrophages or peripheral blood mononuclear cells. Second, although our findings are consistent with differential inflammasome-related responses between ST235 and non-ST235 isolates, inflammasome activation was not directly assessed using specific readouts such as cleaved caspase-1 and caspase-8. Thus, our findings should be interpreted as evidence of differential inflammasome-associated signaling. Third, although pyoverdine was investigated as a potential contributor to the differential host responses, we did not observe significant differences in the effects of ST235- and nonST235-derived pyoverdine under the conditions tested. Nevertheless, this finding does not exclude a potential role of pyoverdine in modulating host responses through concentration-, structural-, or context-dependent mechanisms, which warrants further investigation. Future studies incorporating primary human macrophages, direct measurements of inflammasome activation, and more comprehensive characterization of pyoverdine activity will be important to further define the mechanisms underlying the distinct host responses to ST235 and nonST235 *P. aeruginosa* isolates.

## 4. Methods and Materials

### 4.1. Bacterial Sample Collection

Patients diagnosed with carbapenem-resistant *P. aeruginosa* infection or colonization at Koşuyolu Cardio-Pulmonary Surgery Hospital in Istanbul and Başkent University Hospital in Ankara, Turkey, between July 2017 and December 2018 were included in the study. In total, 44 carbapenem-resistant *P. aeruginosa* isolates were identified by screening 250 Gram-negative infection cases (Supplementary Table S1) and were analyzed using whole-genome sequencing. Among these, the high-risk clone ST235 was detected in 27 isolates. These ST235 isolates carried *FpvAII* and *pvdYII* genes, indicating that these isolates synthesize type II pyoverdines. ST235 isolates also revealed major rearrangements across the pvdD-pvdJ-pvdI loci compared to the PA01 reference. Few SNPs were observed between other PA01 and ST235 pyoverdine biosynthesis genes, including *pvdA*, *pvdF*, *pvdL*, *pvdH*, *pvdG*, and *pvdE*.

### 4.2. Bacterial Growth Conditions, Infection of Cells and İnhibition Assay

All clinical isolates were streaked onto tryptic soy agar (TSA; Sigma-Aldrich, ST. Saint Louis, MO, USA) and incubated overnight at 37 °C. Single colonies were inoculated into tryptic soy broth (TSB) and cultured overnight at 37 °C with shaking, followed by dilution and growth to the mid-logarithmic phase.

THP-1-derived macrophages were infected with bacteria at an MOI of 10 in triplicate. For experiments using antibiotic-killed bacteria, infected cells were centrifuged at 2500 rpm for 7 min and incubated for 3 h. Culture supernatants were serially diluted and plated on TSA, followed by overnight incubation at 37 °C and CFU enumeration. Only extracellular bacterial survival was assessed by CFU enumeration of the culture supernatants; no intracellular bacterial survival assay was performed.

Bacterial growth inhibition was assessed by comparing viable bacterial counts in cultures grown alone or in the presence of THP-1-derived macrophages for 3 h at 37 °C. The inhibition rate was calculated as:

Inhibition rate (%) = [1 − (CFU infected condition/CFU control condition)] × 100.

### 4.3. Cell Culture

THP-1 monocyte cells (ATCC^®^ TIB-202™) were cultured in RPMI 1640 medium supplemented with 10% heat-inactivated fetal bovine serum (FBS), 25 mM HEPES, and 1% penicillin/streptomycin. FBS was heat-inactivated at 56 °C for 30 min before use. THP1-defCASP1 cells (InvivoGen, San Diego, CA, USA), a cell line deficient in caspase-1, were used to investigate the involvement of caspase-1 during *P. aeruginosa* infection. THP1-defCASP1 cells were maintained in the same culture medium supplemented with 200 μg/mL Gold B Hygromycin as the selection antibiotic, according to the manufacturer’s recommendations.

For infection experiments, THP-1 cells were seeded at 5 × 10^5^ cells per well in 24-well, flat-bottom tissue-culture plates. To differentiate THP-1 monocytes into macrophage-like cells, 100 ng/mL phorbol 12-myristate 13-acetate (PMA) was added to the culture medium, and cells were incubated for 24 h. The cells were then washed with phosphate-buffered saline (PBS) and maintained in RPMI 1640 medium supplemented with 10% FBS without PMA for an additional 24 h. Following the differentiation period, the cells were infected with *P. aeruginosa* isolates.

### 4.4. Cell Viability, Cytokine Assays, and Western Blotting

Cell viability was assessed using the alamarBlue Cell Viability Reagent (Thermo Fisher Scientific, Waltham, MA, USA). Following infection, 10 μL of alamarBlue reagent was added to each well and incubated for 1 h according to the manufacturer’s instructions. Fluorescence was measured using a microplate reader at an excitation wavelength of 560 nm and an emission wavelength of 590 nm. Cell viability was calculated based on the fluorescence signal relative to the corresponding uninfected control, which was considered 100% viable. Thus, cell viability (%) was calculated as (fluorescence of infected cells/fluorescence of uninfected control) × 100. All experiments were performed in triplicate. Cell culture supernatants were collected at 3 h post-infection. Cytokine concentrations were determined using human IL-1β (DY201), TNFα (DY210), IL-8 (DY208), and IL-6 (DY206) ELISA kits (R&D Systems, Minneapolis, MN, USA) and a human IL-10 ELISA kit (555157; BD Biosciences, Irvine, CA, USA), following the manufacturers’ protocols. Standard curves were generated using recombinant cytokine standards supplied with each kit, and samples were analyzed in duplicate.

For Western blot analysis, cells were lysed 3 h post-infection using RIPA lysis buffer (Merck, Rahway, NJ, USA) supplemented with 1% 2-mercaptoethanol. Protein concentrations were determined using the BCA/Bradford protein assay (Bio-Rad, Hercules, CA, USA). Equal amounts of protein were separated on %1 SDS-PAGE gels and transferred to PVDF membranes. Membranes were incubated with the following antibodies rabbit anti-phospho-p38 MAPK (Thr180/Tyr182) (D3F9) (cat. Number 9211; Cell Signaling, Danver, MA, USA), rabbit anti-p38 MAPK (cat. Number 9212; Cell Signaling), rabbit anti-phospho-NF-κB p65 (Ser536) (93H1) (cat. Number 3033; Cell Signaling), rabbit anti-NF-κB p65 (D14E12) (cat. Number 8242; Cell Signaling), rabbit anti-active caspase-1 (#4199; Cell Signaling), rabbit anticaspase-1 (cat. Number 3866; Cell Signaling), mouse anti-caspase-8 (8CSP03) (sc-56070; Santa Cruz Biotechnology, Dallas, TX, USA), rabbit anti-caspase-5 (D3G4W) (cat. Number 46680; Cell Signaling), and mouse anti-β-actin (8H10D10) (cat. Number 3700; Cell Signaling). All primary antibodies were diluted 1:1000, followed by HRP-conjugated secondary antibodies: goat anti-rabbit IgG, HRP-linked antibody (cat. Number 7074; Cell Signaling) and horse anti-mouse IgG, HRP-linked antibody (cat. Number 7076; Cell Signaling), and they were diluted to 1:5000. Protein bands were visualized as described previously [17].

### 4.5. RNA Extraction and Quantitative Real-Time PCR (qPCR) Analysis

THP-1 macrophages were infected with ST235 and non-ST235 isolates of *P. aeruginosa* for 3 h. Total RNA was extracted using the RNeasy Mini Kit (Qiagen, Germantown, MD, USA) according to the manufacturer’s instructions and stored at −80 °C until further use. RNA concentration and purity were assessed using a NanoDrop spectrophotometer by measuring the absorbance at 260 nm and the A260/A280 ratio. cDNA was synthesized from the extracted RNA using the iScript cDNA Synthesis Kit (Bio-Rad, Hercules, CA, USA) according to the manufacturer’s instructions, and cDNA concentration was determined using a NanoDrop spectrophotometer prior to qPCR analysis. Quantitative real-time PCR (qPCR) reactions were performed in duplicate in a final volume of 20 μL. Each reaction contained 5 μL of cDNA template (500 ng total cDNA), 10 μL of SYBR Green PCR Master Mix (Bio-Rad, Hercules, CA, USA), and 100 pmol of each forward and reverse primer for the corresponding gene. The thermal cycling conditions were as follows: an initial denaturation at 95 °C for 10 min, followed by 35 cycles of 15 s at 95 °C, 30 s at 55–58 °C, and 30 s at 72 °C. mRNA expression levels of the target genes were normalized to human β-actin, and relative changes in mRNA expression following infection were calculated using the corresponding uninfected control as the reference. The amplification efficiency of each qPCR primer pair was validated using serially diluted cDNA templates, and standard curves were generated for each primer pair. Primer efficiencies were within the acceptable range of 90–110%, with standard curve R^2^ values meeting the recommended criteria. The forward and reverse primer sequences for all target genes are provided in Supplementary Table S2.

### 4.6. Pyoverdine Extraction, NMR Spectroscopy and Structure Determination

Overnight cultures of *P. aeruginosa* were diluted 1:20 in low-iron EMEM (Sigma-Aldrich, Saint Louis, MO, USA) and incubated dynamically at 37 °C for 16 h. Bacterial cells were removed by centrifugation at 3500 rpm for 1 h, and the resulting supernatants were passed through 0.22 μm filters three times. The filtered supernatants were subsequently processed using Amicon^®^ Ultra-15 centrifugal filter units with a 10 kDa molecular weight cutoff (MWCO; Merck, Cat. No. UFC901024). The filtrate was then subjected to ultrafiltration using Amicon Ultra-15 centrifugal filter units with a 3 kDa MWCO to separate pyoverdine from proteins and other high-molecular-weight components. Given the molecular mass of pyoverdine (approximately 1–1.5 kDa), pyoverdine was collected in the filtrate following centrifugation.

The resulting filtrate was further purified by size-exclusion chromatography using an S30 gel filtration column to remove residual low-molecular-weight medium components. A final desalting step was performed using a dedicated desalting column to remove salts and remaining small molecules. The purified pyoverdine-containing fractions were collected and processed for downstream analyses.

The purity of the final pyoverdine preparations was assessed by NMR spectroscopy based on the relative integration of pyoverdine-associated signals and detectable non-pyoverdine signals. The resulting preparations showed >95% purity based on this analysis. Purified pyoverdine samples from ST235 and non-ST235 isolates were lyophilized or SpeedVac-dried and dissolved in 0.6 mL of 90% H_2_O/10% D_2_O at an approximate final concentration of 1 mM. NMR data were acquired under mildly acidic conditions (pH 4.0–5.0), following assessment of sample stability across different pH values and buffer systems.

Spectral quality was initially evaluated using 1D ^1H and 2D ^1H–^1H NOESY experiments. Multidimensional NMR experiments included ^1H–^1H TOCSY, ^1H–^1H NOESY, ^15N–^1H HSQC, ^13C–^1H HMQC, and HMBC. Resonance assignments were performed using CcpNMR, including peak picking, proton assignment, and generation of chemical shift lists (H, C, and N). NOE-derived interproton distance restraints and chemical-shift-derived dihedral angle restraints were generated for structural analysis. Dihedral angles (φ, ψ) were predicted from chemical shift data using TALOS+, with amino acid chemical shift data referenced to the Biological Magnetic Resonance Data Bank (BMRB). Spectra were processed using TopSpin 4.2 (Bruker, Billerica, MA, USA), analyzed using POKY 2.9, and visualized using Mnova 15.1 (Mestrelab Research, Santiago de Compostela, Spain).

### 4.7. M1/M2 Polarization

The M1 phenotype was induced by culturing mature macrophages in 24-well plates (Greiner Bio-One, Kremsmünster, Austria) for 2 days in the presence of 100 ng/mL LPS (Sigma-Aldrich, Saint Louis, MO, USA) + 20 ng/mL recombinant human IFNγ (Sigma-Aldrich, Saint Louis, MO, USA) for M1 polarization. For the last 24 h, the medium was changed to let the cells rest. M2 macrophages were generated using 10 ng/mL IL-10 (ImmunoTools, Friesoythe, Germany). Unactivated macrophages were cultured in medium and left untreated (M0 phenotype). Cells were stained with CD86 (BD Biosciences, Irvine, CA, USA) and CD206 (BD Biosciences, Irvine, CA, USA).

### 4.8. Statistical Analyses

GraphPad Prism 8 (GraphPad Software, San Diego, CA, USA) was used for statistical analyses. Means were compared using either a two-sided unpaired Student’s *t*-test or a Mann–Whitney U test for comparing two groups, with the Shapiro–Wilk test used for verifying normality. For normally distributed data that had unequal variances, Welch’s *t*-test was performed. Simple linear regressions were conducted to determine correlations between percent cell viability and the production of cytokines. Densitometric analyses were completed using ImageJ 1.1 The level of significance was set at *p* < 0.05. The following *p* values were used for figures: * *p* < 0.05, ** *p* < 0.01, *** *p* < 0.001, **** *p* < 0.0001.

## 5. Conclusions

Collectively, our findings reveal that the high-risk ST235 clone possesses a distinct immunomodulatory phenotype that reshapes macrophage responses. Despite promoting a pronounced M1-like polarization, ST235 isolates simultaneously exhibited reduced caspase-1 activity, indicating a finely tuned regulation of inflammasome-associated pathways rather than a generalized pro-inflammatory response. Together with the altered pyoverdine structure and the IL-1β-dominant cytokine profile, these findings suggest that ST235 has evolved a coordinated strategy to manipulate host innate immunity while limiting excessive inflammasome activation that could compromise bacterial persistence. This work identifies immune reprogramming as a previously underappreciated hallmark of the ST235 high-risk clone and provides new mechanistic insight into its exceptional virulence and clinical success.

## Data Availability

The data presented in this study are not publicly available due to ethical and privacy restrictions related to patient-derived clinical isolates and associated data. The datasets are available from the corresponding author upon reasonable request.

## Supplementary Materials

The following supporting information can be downloaded at: https://www.mdpi.com/article/10.3390/ijms27188040/s1.
