# Supplementary material for: Inflammasome Dynamics in the High-Risk Clone ST235 with Altered Pyoverdine Structures
Source: Int J Mol Sci. 2026 Sep 9;27(18):8040. doi: 10.3390/ijms27188040 (PMC13606729; doi:10.3390/ijms27188040)
Supplement: Supplementary file 1 [file ijms-27-08040-s001.zip › ijms-4486616-supplementary.pdf]

Table S1. Clinical and genomic characteristics of the *Pseudomonas aeruginosa* clinical isolates included in this study.

Isolate ID	Source hospital / Region	Biological specimen	Sequence type (ST)	Key SNPs / Genetic characteristics
**K740**	Istanbul	BAL	Non-ST235	—
**K752**	Istanbul	DTA	Non-ST235	—
**K1259**	Ankara	BAL	Non-ST235	—
**K1352**	Ankara	Wound	Non-ST235	—
**K1355**	Ankara	Urinary catheter	Non-ST235	—
**K1357**	Ankara	Urinary catheter	Non-ST235	—
**K1362**	Istanbul	Wound	Non-ST235	—
**K1351**	Istanbul	DTA	Non-ST235	—
**K1364**	Istanbul	BAL	Non-ST235	—
**K1366**	Istanbul	DTA	Non-ST235	—
**K753**	Istanbul	BAL	Non-ST235	—
**K1367**	Ankara	Wound	Non-ST235	—
**K1360**	Ankara	Urinary catheter	Non-ST235	—
**PF125**	Istanbul	BAL	Non-ST235	—
**PF139**	Istanbul	BAL	Non-ST235	—
**PF34**	Istanbul	BAL	Non-ST235	—
**PF33**	Istanbul	BAL	Non-ST235	—
**PF144**	Ankara	Wound	Non-ST235	—
**PF24**	Ankara	Wound	Non-ST235	—
**PF77**	Ankara	Wound	Non-ST235	—
**K1361**	Istanbul	Wound	Non-ST235	—
**K783**	Istanbul	BAL	ST235	pvdA, pvdL
**K982**	Istanbul	BAL	ST235	pvdF
**K989**	Ankara	Catheter	ST235	pvdH, pvdA, pvdF
**K1009**	Istanbul	DTA	ST235	pvdG, pvdE
**K1353**	Istanbul	Urine	ST235	pvdA
**K1359**	Ankara	Wound	ST235	pvdH, pvdL
**K1365**	Istanbul	Urinary catheter	ST235	pvdG, pvdH
**K1363**	Ankara	Urinary catheter	ST235	pvdA, pvdL, pvdE
**K1358**	Ankara	BAL	ST235	pvdG
**K970**	Ankara	Catheter	ST235	pvdE, pvdF
**K704**	Ankara	Catheter	ST235	pvdL

Isolate ID	Source hospital / Region	Biological specimen	Sequence type (ST)	Key SNPs / Genetic characteristics
**PF247**	Ankara	Blood	ST235	pvdA, pvdE, pvdF
**PF453**	Istanbul	DTA	ST235	pvdH, pvdE
**PF103**	Ankara	Blood	ST235	pvdF
**PF451**	Istanbul	Blood	ST235	pvdA
**PF197**	Istanbul	Blood	ST235	pvdA, pvdE, pvdF
**PF312**	Ankara	Blood	ST235	pvdA
**K741**	Istanbul	BAL	ST235	pvdG, pvdH, pvdE
**K748**	Istanbul	DTA	ST235	pvdA
**PF63**	Ankara	BAL	ST235	pvdH

Abbreviations: ST, sequence type; SNP, single-nucleotide polymorphism; MIC, minimum inhibitory concentration; BAL, bronchoalveolar lavage; DTA, deep tracheal aspirate.

Table S2. Primers for cytokine and reference gene targets

Gene Target	Primer	Sequence (5′–3′)
**Human IL-1β**	Forward	CCACAGACCTTCCAGGAGAATG
	Reverse	GTGCAGTTCAGTGATCGTACAGG
**Human TNF**	Forward	CTCTTCTGCCTGCTGCACTTTG
	Reverse	ATGGGCTACAGGCTTGTCACTC
**Human IL-8**	Forward	GAGAGTGATTGAGAGTGGACCAC
	Reverse	CACAACCCTCTGCACCCAGTTT
**Human IL-10**	Forward	TCTCCGAGATGCCTTCAGCAGA
	Reverse	TCAGACAAGGCTTGGCAACCCA
**Human β-actin**	Forward	GACTTCGAGCAAGAGATGGC
	Reverse	CACAGGACTCCATGCCCAG

SUPPLEMENTARY FIGURE

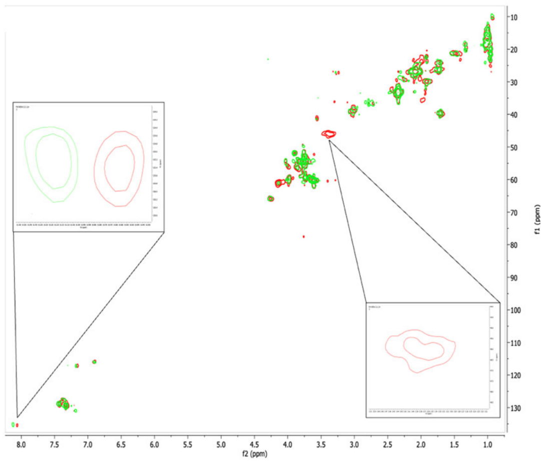

Supplementary figure S1: Overlay of the 2D ^1H–^13C HMQC spectra of pyoverdine extracted from PAO1 (red) and ST235 (green) isolates, highlighting distinct chemical shift differences between the two samples.
